# Longitudinal bone loss in the paretic leg and its contributing factors in individuals with chronic stroke: a 2-year prospective cohort study

**DOI:** 10.1007/s11657-025-01541-4

**Published:** 2025-08-06

**Authors:** Huixi Ouyang, Tiev Miller, Ling Qin, Michael T. C. Ying, Vivian W. Y. Hung, Thomas W. H. Leung, Marco Y. C. Pang

**Affiliations:** 1 School of Biomedical Engineering, Guangzhou Medical University, Guangzhou, China; 2 Department of Rehabilitation Sciences, The Hong Kong Polytechnic University, Kowloon, Hong Kong; 3 International Collaboration On Repair Discoveries, University of British Columbia, Vancouver, BC Canada; 4 Division of Physical Medicine and Rehabilitation, Department of Medicine, University of British Columbia, Vancouver, BC Canada; 5 Department of Orthopaedics and Traumatology, Bone Quality and Health Centre and Center of Aging in Musculoskeletal Degeneration and Regeneration, The Chinese University of Hong Kong, Sha Tin, Hong Kong; 6 Department of Health Technology and Informatics, The Hong Kong Polytechnic University, Kowloon, Hong Kong; 7 Department of Medicine & Therapeutics, Faculty of Medicine, The Chinese University of Hong Kong, Sha Tin, Hong Kong

**Keywords:** Bone, Stroke, Tomography, Blood circulation, Walking speed, Physical fitness, Lower extremity

## Abstract

**Summary:**

Post-stroke fracture risk necessitates investigation of bone properties and contributing factors. The decline in paretic tibia failure load post-stroke was attributed to decreased trabecular bone density and thickness at 2-year follow-up. Less decline in bone strength was associated with better leg blood flow, walking speed, strength, and activity at baseline.

**Purpose:**

To delineate long-term changes in distal tibia bone properties after stroke and identify their associated factors.

**Methods:**

High-resolution peripheral quantitative computed tomography (HR-pQCT) scans of the bilateral distal tibia were performed in 46 chronic stroke participants (age, 60.4 ± 7.8 years; post-stroke onset, 6.3 ± 4.2 years) and 45 controls (age, 57.7 ± 6.3 years) at baseline and 2 years later. We measured the change in the estimated failure load (indicator of bone strength), volumetric bone mineral density (vBMD), geometry, and microstructure. Blood flow volume of the popliteal artery, muscle strength, sensory function, and gait speed were also assessed.

**Results:**

In the paretic leg of stroke participants, a significant decline in estimated failure load was observed (− 3.39%, *p* < 0.01), which was greater than that of the non-paretic side (− 1.93%, *p* < 0.01) and controls (− 1.89 to − 2.18%, *p* < 0.05). The deterioration in estimated failure load was accompanied by a decline in trabecular vBMD and thickness. Greater arterial blood flow, higher walking velocity, better muscle strength, and higher physical activity level at baseline at 2-year follow-up portended less decline in estimated failure load.

**Conclusions:**

During the 2-year follow-up, there was a decline in estimated failure load of the paretic distal tibia among people with chronic stroke, attributed to a decreased trabecular density and thickness. Greater decline in estimated tibial bone strength was associated with lower arterial blood flow volume and motor function on the paretic side.

**Supplementary Information:**

The online version contains supplementary material available at 10.1007/s11657-025-01541-4.

## Introduction

In the era of population aging, stroke is a major public health concern. It is estimated that 1 in 12 patients will experience a femoral fracture within 5 years after stroke [1]. Post-stroke fracture further undermines long-term daily function despite prolonged rehabilitation [2]. Although fracture prevention is of paramount importance, stroke-related factors precipitating the diminution of bone integrity over time remain relatively understudied.

Secondary osteoporosis increases fracture risk substantially after stroke [3]. Previous studies using dual-energy x-ray absorptiometry (DXA) revealed a 10–12% reduction in areal bone mineral density (aBMD) of the proximal femur on the paretic side within the first year after stroke [4, 5]. The rate of bone loss was approximately 10 times greater than that of healthy counterparts [6]. Changes in bone properties are also evident during the chronic stage of stroke recovery (mean post-stroke onset: 4 years). Using peripheral quantitative computed tomography (pQCT), a remarkable decline in trabecular volumetric bone mineral density (vBMD) of the paretic distal tibia was observed after only 1 year [7].

However, previous DXA and pQCT studies were unable to assess bone microstructure (e.g., cortical thickness in distal limbs), a critical determinant of overall bone strength [4, 5, 7]. The microarchitecture of the tibia can discriminate fragility fracture status in postmenopausal women independent of DXA-derived aBMD [8]. The Bone Microarchitecture International Consortium (BoMIC), which includes data from eight cohorts (*n* = 7254), reported that total vBMD or trabecular vBMD, tibial cortical vBMD, and cortical thickness improved fracture prediction in elderly individuals when compared with femoral neck aBMD or FRAX scores alone [9]. Therefore, HR-pQCT is considered to be superior to DXA in fracture discrimination [10]. Moreover, human cadaver studies have shown that the estimated failure load (an HR-pQCT-based bone strength index derived from the micro finite element analysis) is more related to the actual failure load than other bone variables measured by pQCT (e.g., vBMD or cortical thickness) [11]. In predicting facture risk among older adults, estimated failure load is also superior to other bone variables (e.g., vBMD) [9] and cannot be generated from DXA and pQCT scans. Studies involving the use of high resolution-pQCT (HR-pQCT) to evaluate longitudinal changes in bone microstructure among people with chronic stroke are currently lacking.

In parallel, the determinants of progressive bone deterioration after stroke are understudied. Mechanical stress from ground reaction forces and muscle contractions during physical activities are considered to be beneficial in either maintaining or improving bone integrity [12, 13]. Previous longitudinal studies involving the use of first-generation pQCT or DXA scans found an association between muscle strength, motor recovery [7], spasticity [14], and bone loss during the chronic stage of stroke. However, the relative contribution of these factors to changes in bone health remains unknown. Identifying clinically relevant predictors of bone loss is critical for designing intervention programs that specifically target bone health post-stroke. As the development of osteoporosis after stroke is likely multi-factorial in etiology, a comprehensive prediction model including demographic factors (e.g., age, sex) is warranted.

Thus, this longitudinal cohort study aimed to (1) assess the 2-year change in tibia estimated bone strength, density, geometry, and microstructure using HR-pQCT; and (2) identify predictors of the changes in tibia estimated bone strength in people with chronic stroke.

## Methods

### Study design

This was a 2-year prospective cohort study involving individuals with chronic stroke and control participants.

### Subject recruitment

We recruited individuals with chronic stroke from community self-help groups and the general public between October of 2018 and September of 2021. The baseline findings have been published [40]. Inclusion criteria were as follows: (1) a history of stroke with unilateral hemiparesis; (2) age ≥ 18 years; (3) stroke onset > 1 year; (3) a modified Rankin Scale (MRS) of 2–5. Exclusion criteria were as follows: (1) concurrent neurological conditions; (2) receptive aphasia; (3) recurrent strokes; (4) other conditions with a substantial effect on bone health; (5) receiving pharmacological treatment for osteoporosis; (6) an established diagnosis of osteoporosis prior to index stroke; (7) fragility fractures prior to stroke; and (8) metallic implants in the scanned limb. A control group with no history of stroke was recruited from the community by convenience sampling using the same inclusion and exclusion criteria.

Sample size estimations were based on an alpha of 0.05 (two-tailed) and a power of 0.8. Assuming a 15% attrition rate, we aimed to recruit a minimum of 54 individuals for each group. A detailed description of the sample size estimation is provided in Electronic Supplementary Material (ESM) 1.1.

### Demographic information

Basic demographic data were obtained from participant medical records (stroke group) and questionnaires (control group). Bone scans and other clinically relevant measurements were performed within a week at the initial assessment (T1) and again at the 2-year follow-up (T2). Previous studies showed that the temporal changes in some bone variables within a 1-year period were quite modest [7, 15]. Thus, to observe a detectable and clinically significant change recommended by The National Osteoporosis Foundation [16], this study used a 2-year assessment interval. The HR-pQCT scan was performed by a technician at a local bone imaging center with extensive experience in osteoporosis research. Two other qualified researchers with more than 5 years of research experience, and one research assistant performed the other clinical measurements. All measurements, except the stroke-specific assessments, were administered to both groups.

### HR-pQCT scan protocol

Although the proximal femur is the most common site of post-stroke lower-limb fracture [17], it cannot be scanned using HR-pQCT due to its central location. Therefore, the distal tibia was chosen as the measurement site for its high correlation with femoral bone properties [18]. The vBMD, cross-sectional geometry, and microstructural properties of the bilateral distal tibiae were measured using a second-generation HR-pQCT scanner (XtremeCT II, Scanco Medical AG, Brüttisellen, Switzerland). All HR-pQCT outcomes are listed in Electronic Supplementary Material (ESM) Table 2.1. The scan region was fixed at 22.0 mm (distal tibia) proximal from the mid-joint line and was 10.2 mm in length. In the event Grade 4 or 5 motion artifacts were identified during scanning, the scan was repeated up to two times. All images were graded for quality by a single operator according to a visual grading system proposed by Pialat et al. [19]. The entire volume of interest (VOI) was separated into cortical and trabecular components using a fully automated cortical compartment segmentation technique [20]. The contour was carefully checked and corrected, if necessary, by the same operator who graded the image quality. The VOI between baseline and 24-month HR-pQCT scans was matched by 2D registration.

Analyses of the three-dimensional (3D) scan data were conducted using Image Processing Language (IPL v5.08b; Scanco Medical AG, Brüttisellen, Switzerland). All micro finite element (μFE) analyses were performed using the FE-solver included in the built-in IPL software (IPL-FE v1.15, Scanco Medical AG) [21]. μFE analyses were performed by converting the binary image data to a mesh of isotropic brick elements. A linear high-friction compression model was applied with a Young’s modulus of 10 GPa and a Poisson’s ratio of 0.3 assigned. The estimated failure load (N) was calculated based on the assumption that bone failure occurred if > 2% of the elements were strained beyond 0.7% [22]. The reproducibility and Least Significant Change (LSC) of the HR-pQCT variables have been established (ESM 2.1).

### Other assessments

*Muscle strength:* A dynamometer system (Humac Norm Systems, Stoughton, MA, USA) was used to measure isometric peak torque (N/m) of the ankle plantar flexor muscles at 0° [23, 24]. Tests were performed three times with a 1-min rest interval between attempts to calculate the average strength value.

#### Walking status

The 10-m walk test was implemented at a safe maximal speed and demonstrated excellent test–retest reliability [intra-class correlation coefficient (ICC) = 0.97) [25]. The Functional Ambulation Categories (FAC) were also used to classify the level of walking independence.

#### Sensory function

The touch pressure threshold of the foot plantar surface was tested using Semmes–Weinstein monofilaments (SWMT) [26]. In individuals with chronic stroke, SWMT grade scores have demonstrated substantial to perfect intra-rater agreement (Cohen’s kappa values: thumb = 0.89) [26].

#### Physical activity level

The Physical Activity Scale for the Elderly (PASE) [27] was used to evaluate physical activity level (score range, 0 to 400). Chinese version of the PASE has demonstrated good test–retest reliability in healthy older adults (ICC = 0.81)] [27].

#### Arterial blood flow

Blood flow velocity (cm/s) and volume (mL/min) of the popliteal artery were measured using Doppler ultrasound (AixPlorer, Supersonic Imagine, Aix-en-Provence, France) coupled with a linear transducer (4–15 MHz) [28]. Within the same image, the arterial diameter (cm) was also measured. Doppler ultrasound settings were standardized and set for high sensitivity, a medium wall filter, a pulsed repetition frequency of 4162 Hz, and medium persistence. The sample volume was standardized at 1 mm, and Doppler angle correction was applied. A 15-min rest period was required before each examination. Three consistent spectral waveform cycles were used to calculate the average measures. To examine the reliability of vascular measures, we conducted a pilot trial involving 15 individuals with chronic stroke (ESM 2.2) and found good to excellent inter-rater (ICC = 0.85–0.93) and intra-rater reliability (ICC = 0.90–0.98).

### Stroke-specific assessments

#### Motor recovery

He Fugl–Meyer Motor Assessment (FMA) was used to determine the degree of hemi-paretic lower limb motor recovery (range, 0–34). The FMA assesses movement quality, movement coordination, and reflex action of the hip, knee and ankle. The interrater reliability of the FMA was excellent (ICC = 0.96) [29].

#### Spasticity

Spasticity of the ankle plantar flexors was measured using the Composite Spasticity Scale (CSS; score range, 1–16) [30]. The test–retest reliability of the CSS was also excellent (ICC = 0.97) [30].

### Statistical analysis

There was no missing data for participants who completed the HR-pQCT scans at 2-year follow-up. Descriptive statistics were used to summarize and tabulate the data. An independent samples Student’s *t* test, Mann–Whitney *U* test, or chi-square (*χ*^2^) test was used to compare measured variables between the stroke and control groups, depending on whether the data fulfilled the criteria for parametric statistics.

Generalized estimating equations (within-subject factors: side and time) were used to determine if there was an interaction effect between the two factors in two groups. Separate paired *t* or Wilcoxon signed-rank tests were performed to compare baseline and follow-up data as necessary. Independent *t* or Mann–Whitney *U* test were used to compare between-group differences in relative change scores [(T2-T1)/T1] for each variable, with negative values indicating a decrease from baseline to follow-up. Bonferroni correction was applied for post hoc comparisons.

Pearson’s correlations were used to examine associations between the change in estimated failure load (from baseline to 24-month follow-up) and impairment variables measured at baseline (e.g., muscle strength and spasticity). Variables showing correlation at a significance level of *p* < 0.1 were chosen as independent (predictor) variables in subsequent regression analyses to identify baseline modifiable predictors of the change in paretic side estimated failure load for the stroke group, while adjusting for potentially confounding factors influencing bone health (e.g., age, sex, post-stroke duration).

A principal component analysis (PCA) with varimax rotation was then performed to extract non-modifiable factors to be used as independent variables in the multivariable regression analysis. In considering the current sample size and to avoid overfitting in the regression analysis, a separate PCA (i.e., dimension reduction) with varimax rotation was performed to identify potentially modifiable factors to be used as independent variables in the event three or more variables showed significant bivariate correlations with the dependent variable. Kaiser–Meyer–Olkin tests were used to ensure sampling adequacy for the PCA at a threshold value ≥ 0.5. PCA-derived modifiable, non-modifiable and other factors (e.g., total number of medications) were then entered into the model using a hierarchical regression series procedure [31]. In order to improve the prediction model and adjust for potential variation in the location of the scan region, the percentage change in height was also added to determine whether this would affect the model.

## Results

### Participant characteristics

One hundred and twenty-eight individuals (64 people with stroke and 64 controls) were recruited. A study flowchart is provided in ESM Fig. 2.1. Forty-six individuals with stroke and 45 controls who completed the 2-year follow-up were included in the final analysis. Participant characteristics are summarized in Table 1.

**Table 1:** Demographic information

Variable	Stroke (*n* = 46)	Control (*n* = 45)	*p*
**Basic demographics**			
Age (year)	60.4 ± 7.8	57.7 ± 6.3	0.074
Stroke duration (years)	6.3 ± 4.2	NA	NA
Body mass index (kg/m^2^)	24.2 ± 3.1	23.4 ± 2.8	0.056
Dominant leg (equivalent/right/left, *n*)	1/44/1	0/44/1	1.000
Sex (female/male, *n*)	21/25	17/28	0.446
Postmenopausal women (yes/no, *n*)	18/28	15/30	0.662
Walking aids (none/outdoor cane or stick/outdoor quadripod/both indoor and outdoor cane or stick/both indoor and outdoor quadripod/both indoor and outdoor wheelchair, *n*)	11/27/1/1/4/2	45/0/0/0/0/0	< 0.001
Oxfordshire Classification (anterior circulation syndrome/partial anterior circulation syndrome/lacunar syndrome/hemorrhage, *n*)	7/16/7/16	NA	NA
Alcohol history (non-drinker/drinking history, *n*)	37/9	28/17	0.055
Smoking history (non-smoker/smoking history, *n*)	36/10	33/12	0.583
Functional Ambulation Category (dependent/dependent with supervision/independent on level surfaces only/independent, *n*)	1/2/4/39	NA	NA
Modified Rankin Scale (1/2/3, *n*)	2/31/13	NA	NA
**Medications/supplements**			
Calcium (no supplementation/yes, *n*)	1	3	0.361
Vitamin D (no supplementation/yes, *n*)	2	0	0.495
Antihypertensive agents, *n*	27	12	0.002
Anticoagulants, *n*	16	0	< 0.001
Anticonvulsive agents, *n*	4	0	0.117
Hypolipidemic agents, *n*	27	7	< 0.001
Hypoglycemic agents, *n*	7	4	0.354
Antidepressants, *n*	5	0	0.056
Proton pump inhibitors, *n*	20	0	< 0.001
Total number of medications	4.4 ± 3.4	0.8 ± 1.3	< 0.001
**Co-morbid conditions**			
Hypertension, *n*	25	16	0.072
Diabetes, *n*	10	6	0.292
High cholesterol, *n*	15	4	0.005
Total number of comorbidities	1.3 ± 1.4	0.7 ± 1.0	0.016

Mean ± SD. *NA* not applicable

### Changes in bone outcomes

**Fig. 1:**
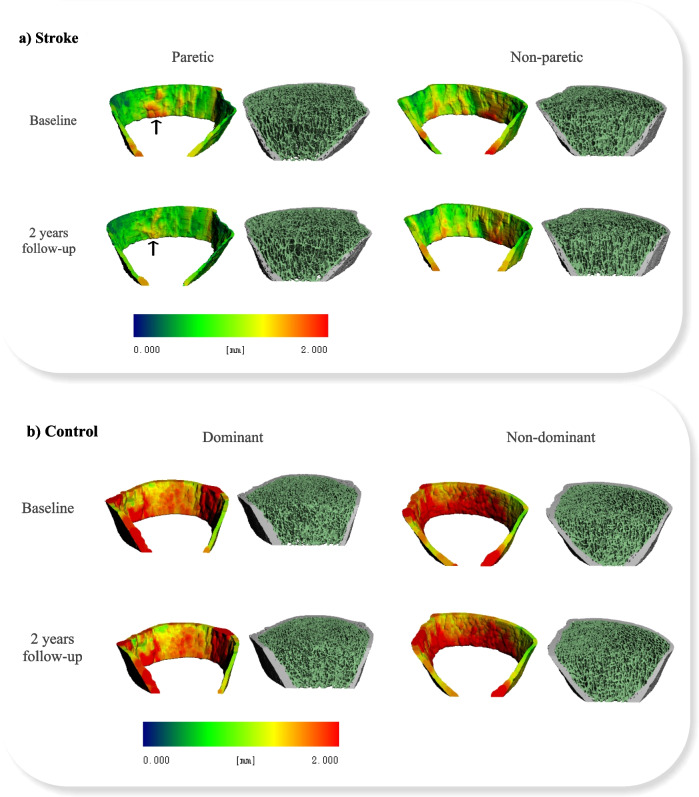
HR-pQCT images of the distal tibia obtained from a participant with stroke (**a**) and a control participant (**b**). The decrease in cortical thickness was more apparent on the paretic side of the participant with stroke over the 2-year follow-up period, as indicated by the diminished area in red (black arrow). Compared to baseline, trabecular network density also decreased on the paretic side at 2-year follow-up

The common region after image matching between baseline and 2-year follow-up ranged from 85 to 99% in the stroke group and 93 to 99% in the control group. The bone images from two representative participants are shown in Fig. 1.

Significant reductions in cortical vBMD and estimated failure load were observed for both sides in both groups (*p* < 0.0125). However, significant reductions in cortical thickness were only observed in the stroke group on both sides (*p* < 0.0125). Furthermore, significant reductions in trabecular vBMD, trabecular thickness, and cortical area only occurred on the paretic side in the stroke group (*p* < 0.0125) (Table 2).

**Table 2:** HR-pQCT tibia variables for the stroke and control groups

	Stroke group (*n*=46)						Control group (*n*=45)					
	Baseline		2-year follow up		*p*		Baseline		2-year follow up		*p*	
	P	NP	P	NP	P	NP	ND	D	ND	D	ND	D
Total vBMD (mg HA/cm^3^)	263.22± 70.01	286.02± 58.33	258.34 ± 71.65	283.20±59.05	< 0.001*	0.002*	278.95±54.16	279.85±55.73	276.32 ± 54.69	276.48±55.31	0.013	0.017
Total area (mm^2^)	651.14±125.14	658.15± 124.26	656.34 ± 125.61	663.42±124.76	< 0.001*	< 0.001*	708.65±141.22	710.14±138.65	714.07 ± 141.77	715.58±139.18	< 0.001*	< 0.001*
Trabecular area (mm^2^)	545.75± 126.51	539.14± 122.34	547.41 ± 126.18	540.07± 121.80	0.007*	0.085 ^a^	583.66± 135.57	585.54 ± 135.46	583.55 ± 134.95	586.03±134.24	0.836	0.376
Trabecular vBMD (mg HA/cm^3^)	147.05±41.16	152.61±35.68	145.80 ± 42.09	152.87 ± 35.87	0.024	0.502	143.01 ± 34.96	143.80 ± 32.67	142.80 ± 35.74	143.96± 33.04	0.534	0.721
Trabecular number (1/mm)	1.145 ± 0.173	1.147 ± 0.153	1.143 ± 0.186	1.151 ± 0.181	0.792	0.662	1.083 ± 0.172	1.098 ± 0.168	1.079 ± 0.186	1.096 ± 0.173	0.324	0.578
Trabecular thickness (mm)	0.253 ± 0.025	0.255 ± 0.021	0.251 ± 0.024	0.255 ± 0.020	0.012*	0.261	0.253 ± 0.021	0.252 ± 0.021	0.253 ± 0.021	0.253 ± 0.021	0.113	0.010*
Trabecular separation (mm)	0.858 ± 0.148	0.852 ± 0.137	0.868 ± 0.185	0.860 ± 0.165	0.302^a^	0.646^a^	0.911 ± 0.183	0.891 ± 0.152	0.928 ± 0.240	0.898 ± 0.162	0.018^a^	0.023
Trabecular inhomogeneity (mm)	0.350 ± 0.06	0.352 ± 0.06	0.353 ± 0.07	0.357 ± 0.07	0.085	0.105	0.371 ± 0.07	0.361 ± 0.07	0.372 ± 0.07	0.366 ± 0.08	0.721	0.001*
Cortical area (mm^2^)	110.56 ± 32.82	124.18 ± 29.75	108.92 ± 33.75	123.35 ± 30.49	0.007*	0.137	130.36 ± 24.12	129.99 ± 24.23	130.52 ± 25.22	129.55± 24.91	0.760	0.429
Cortical vBMD (mg HA/cm^3^)	813.32 ± 87.54	847.81 ± 69.42	802.29 ± 82.98	837.88 ± 66.13	< 0.001*	< 0.001*	870.48 ± 59.82	871.50 ± 59.55	858.41 ± 56.95	857.69± 57.13	< 0.001*	< 0.001*
Cortical perimeter (mm)	100.01 ± 9.37	100.55 ± 9.36	100.06 ± 9.46	100.73 ± 9.38	0.489	0.011*	103.82 ± 10.42	103.83 ± 10.19	104.04 ± 10.55	105.33± 13.43	0.001*	0.001*^a^
Cortical porosity (%)	0.038 ± 0.017	0.035 ± 0.014	0.041 ± 0.017	0.037 ± 0.015	0.017	0.046	0.033 ± 0.015	0.031 ± 0.015	0.036 ± 0.016	0.034 ± 0.016	< 0.001*	0.004*
Cortical thickness (mm)	1.326 ± 0.399	1.474 ± 0.355	1.305 ± 0.414	1.457 ± 0.358	0.011*	0.009*	1.481 ± 0.283	1.469 ± 0.309	1.475 ± 0.291	1.459 ± 0.300	0.320	0.137
Failure load (N)	8285.13 ± 2390.70	9278.31 ± 2182.68	8037.26 ± 2434.01	9102.36 ± 2165.22	< 0.001*	< 0.001*	9754.45 ± 2059.24	9813.21 ± 2015.50	9596.26±2140.51	9624.58±2102.79	< 0.001*	< 0.001*

*P* paretic side, *NP* non-paretic side, *ND* non-dominant side, *D* dominant side, *vBMD* volumetric bone mineral density

* *p* < 0.0125 (paired *t* test with Bonferroni correction); ^a^non-parametric test

Significantly greater decline in estimated failure load was observed for the paretic side (− 3.39%) than non-paretic side (− 1.93%) (*p* = 0.002). The decline in estimated failure load of the paretic side for the stroke group was insignificant, and only marginally greater than the non-dominant side of controls (− 1.89%, *p* = 0.019). Additionally, declines in trabecular vBMD and thickness were significantly greater for the paretic than non-paretic side (*p* < 0.0125), whereas only the decline in the trabecular thickness was significantly greater for the stroke group (paretic, − 0.58%) than the control group (non-dominant, 0.28%; *p* = 0.005). The decline in cortical area was also marginally insignificant between groups (*p* = 0.013) (Table 3).

**Table 3:** The relative change in tibia bone variables over the 2-year follow-up period

	Stroke group (*n* = 46)		*Between-side*	Control group (*n* = 45)		*Between -side*	*Between-group*	
	Paretic	Non-paretic	*p*	Non-dominant	Dominant	*p*	*p (*paretic)	*p (*non-paretic)
Total vBMD (mg HA/cm^3^)	2.14% ± 3.24%	1.02% ± 2.20%	0.008‡	0.99% ± 2.43%	1.17% ± 2.97%	0.666	0.038	0.659
Total area (mm^2^)	0.81% ± 0.08%	0.81% ± 0.07%	0.903	0.78% ± 0.08%	0.78% ± 0.08%	0.689	0.047	0.050
Trabecular area (mm^2^)	− 0.32% ± 0.74%	− 0.20% ± 0.76%	0.232	0.00% ± 0.68%	− 0.14% ± 0.81%	0.133	0.036	0.207
Trabecular vBMD (mg HA/cm^3^)	1.18% ± 3.65%	− 0.15% ± 2.06%	0.001‡	0.36% ± 2.21%	− 0.05% ± 2.44%	0.190	0.294	0.831
Trabecular number (1/mm)	0.33% ± 4.29%	− 0.18% ± 5.78%	0.476	0.69% ± 3.86%	0.23% ± 2.10%	0.937	0.450	0.370
Trabecular thickness (mm)	0.58% ± 1.58%	− 0.29% ± 1.40%	0.002‡	− 0.28% ± 1.24%	− 0.60% ± 1.50%	0.233	0.005§	0.299
Trabecular separation (mm)	− 0.83% ± 4.51%	− 0.68% ± 5.52%	0.202	− 1.24% ± 4.34%	− 0.69% ± 1.82%	0.913	0.384	0.319
Trabecular inhomogeneity (mm)	− 0.78% ± 2.21%	− 0.51% ± 2.30%	0.425	− 0.73% ± 1.93%	− 1.07% ± 1.56%	0.036	0.894	0.178
Cortical area (mm^2^)	1.83% ± 4.92%	0.77% ± 3.58%	0.069	− 0.01% ± 2.74%	0.41% ± 2.83%	0.137	0.013	0.306
Cortical vBMD (mg HA/cm^3^)	1.28% ± 2.36%	1.13% ± 1.65%	0.517	1.36% ± 1.86%	1.55% ± 2.19%	0.143	0.617	0.943
Cortical perimeter ((mm)	− 0.04% ± 0.54%	− 0.18% ± 0.42%	0.032	− 0.20% ± 0.32%	− 1.43% ± 8.19%	0.643	0.108	0.566
Cortical porosity (%)	− 12.04% ± 24.12%	− 7.89% ± 19.71%	0.238	− 9.79% ± 15.79%	− 13.59% ± 27.62%	0.316	0.601	0.880
Cortical thickness (mm)	1.95% ± 5.29%	1.22% ± 3.37%	0.714	0.46% ± 2.50%	0.57% ± 2.76%	0.576	0.228	0.322
Failure load (N)	3.39% ± 3.46%	1.93% ± 2.48%	0.002‡	1.89% ± 2.44%	2.18% ± 2.84%	0.312	0.019	0.703

relative change = (T _follow up_ -T _baseline_)/T _baseline_; negative values suggest decline from baseline to follow-up

‡*p* < 0.0125, significant difference between two sides (paired *t* test)

§*p* < 0.0125, significant difference between two groups (independent *t* test, dominant [controls] vs. non-paretic sides, the non-dominant [controls]vs paretic sides)

### Variables at baseline that were associated with changes in failure load

PCA-derived non-modifiable factors (Factor 1: age, sex and smoking history; Factor 2: stroke duration and alcohol history), total number of medications (Factor 3), and potentially modifiable factors (Factor 4: gait velocity, muscle strength and PASE; Factor 5: blood flow volume) are summarized in ESM Tables 2.8 and 2.9. Among modifiable factors, higher walking speed (*r* = 0.30, *p* = 0.043) and greater blood flow volume (*r* = 0.36, *p* = 0.013) *at baseline* were associated with less of a decline in estimated failure load. In addition, greater physical activity level (*r* = 0.27, *p* = 0.071) and greater muscle strength (*r* = 0.28, *p* = 0.059) also showed a marginally significant association with less change in estimated failure load (ESM Table 2.10). After accounting for relevant non-modifiable factors and total number of medications, both Factor 4 (i.e., baseline walking speed, muscle strength and PASE, Beta = 0.37, *p* = 0.012) and Factor 5 (i.e., baseline blood flow volume, Beta = 0.34, *p* = 0.021) remained significantly associated with the change in estimated failure load, explaining 12% and 12% of its variance, respectively (Table 4).

**Table 4:** Regression analysis: associations between baseline principal components and the %change in paretic tibia estimated failure load

Independent variables	Model summary		Δ*F*	*p* (ΔF)	Standardized regression coefficients	
	*R*^2^	Δ*R*^2^			Beta	*p*
**Model 1**						
PCA Factor 1	0.02	0.02	0.85	0.361	− 0.14	0.361
PCA Factor 2	0.05	0.03	1.49	0.229	0.18	0.231
PCA Factor 3	0.06	0.01	0.55	0.463	0.11	0.463
**Model 2**						
PCA Factor 4	0.18	0.12	5.73	0.021*	0.34	0.015†
PCA Factor 5	0.30	0.12	6.88	0.012*	0.37	0.012†

*R*^*2*^ total variance, *ΔR*^*2*^ additional predictor variance, *ΔF F*-value change, *Beta* standardized regression coefficient, *CI* confidence interval, *%change* percent change, *PCA* principal component analysis, *vBMD* volumetric bone mineral density

Factors represent variables included in the exploratory dimension reduction (i.e., principal component analysis)

Variables entered: age, alcohol history, blood flow volume, body mass index, gait speed, muscle strength, Physical Activity Scale for the Elderly, sex, smoking history, stroke duration, total number of comorbidities, total number of medications

Variables excluded (Kaiser–Meyer–Olkin < 0.5): body mass index, total number of comorbidities

Principle component analysis-derived factors:

Factor 1 = Age, sex, smoking history

Factor 2 = Stroke duration, alcohol history

Factor 3 = Total number of medications

Factor 4 = Gait speed, muscle strength, Physical Activity Scale for the Elderly

Factor 5 = Blood flow volume

*p ≤ 0.05 statistically significant *F*-value change

†*p* ≤ 0.05 Statistically significant predictor

Adding % change in height did not improve the two models

## Discussion

This study examined longitudinal changes in distal tibia bone properties and identified clinically relevant predictors of estimated bone strength in a cohort of individuals with chronic stroke. Overall, a decline was observed in both cortical and trabecular bone variables in the paretic distal tibia of people with chronic stroke. The decrease in estimated failure load was accompanied by a decline in cortical vBMD and cortical thickness. Baseline arterial blood flow volume and motor function were associated with the change in estimated failure load during the 2-year follow-up period.

### Changes to bone properties in the stroke group

In the current study, the decline in cortical thickness was only observed in the stroke group despite no significant difference between the paretic and non-paretic sides. The reduction in cortical vBMD was comparably more pronounced for both sides in both stroke and control groups, possibly reflecting an age-related diminution in cortical bone volume. On the other hand, the decline in trabecular vBMD and thickness was significantly greater for the paretic than non-paretic side. Additionally, the decline in trabecular thickness was also significantly greater for the stroke group than the control group, while the decline in cortical area and estimated failure load were only marginally insignificant between groups. This suggests that although both cortical and trabecular bone loss at the distal tibia appear to continue during the chronic stage of stroke, the reduction in trabecular thickness emerged as the only significant group-based disparity among bone parameters over the follow-up period. More studies involving a longer follow-up with larger cohorts are needed to examine potential group-dependent effects across bone parameters.

The degradation in cortical thickness may be due to endocortical trabecularization, during which intracortical and endocortical remodeling erode cortical bone [32]. This was supported by our findings which show that decreased cortical thickness was accompanied by an increased trabecular area, with no change in cortical perimeter on the paretic side. This differs from the aging process, during which decreased cortical thickness occurs together with an enlarged cross-sectional area through periosteal remodeling, as part of a compensatory mechanism to partially counteract the detrimental effect of bone loss on overall bone strength [33].

### Percentage change in bone properties, failure load, and fracture risk

Available evidence suggests that a small reduction in bone mass leads to a substantial increase in fracture risk. According to a meta-regression analysis of 38 placebo-controlled trials, a 2% or 6% improvement in total hip BMD may lead to a 16% or 40% reduction in hip fracture risk [34]. In this study, there was an average decrease of 1.28% in cortical vBMD, a 1.18% reduction in trabecular vBMD, and a 3.4% decrease in estimated failure load of the distal tibia by the 2-year follow-up period in the stroke group. The tibial failure load estimated by HR-pQCT is an important variable, because its ability to predict fracture has been established in postmenopausal women (odds ratio = 3.85; 95% CI, 1.47 to 10.0) [35] and older adults (hazard ratio = 2.46; 95% CI, 1.81 to 3.34) [9]. Taken together, it is reasonable to presume that fracture risk is substantially increased as a result in individuals with chronic stroke.

### Blood flow as a factor associated with bone loss

We found that baseline blood flow volume was associated with the reduction in estimated failure load in the hemi-paretic distal tibia. A previous 1-year longitudinal study of individuals with chronic stroke also found a negative relationship between vascular elasticity index and the decline in cortical thickness (measured by pQCT) in the radius diaphysis [15]. The relevance of vascular function in bone health has also been demonstrated in older adults. A large cohort study demonstrated a significant association between the arterial stiffness index and bone quality (i.e., bone speed of sound measured using ultrasound, m/s) among the general population aged 40 to 69 (median = 58, IQR = 50 to 63) [36]. As bone is a highly vascularized structure, better vascular function may have a protective effect on maintaining bone tissue integrity. According to a large cross-sectional study of menopausal women (*n* = 386), adipokines (e.g., adiponectin and vaspin) may mediate the association between arterial stiffness and osteoporosis [37]. However, the mechanisms underlying this relationship are still uncertain and require more research.

### Walking speed, muscle strength, and physical activity as factors associated with bone loss

In the present study, higher walking speed, greater leg strength, and higher physical activity level at baseline were found to be associated with less of a decline in estimated failure load. A faster walking speed is indicative of better mobility, motor function and is related to higher ground reaction forces generated during walking [38] and more frequent ambulatory activity [39, 40]. These factors may contribute to greater bone tissue loading, thereby providing more protection against deleterious changes in bone properties.

Similar results were found during the early stages of stroke recovery. The recovery of independent walking has been found to be associated with reduced bone loss in the distal tibia (vBMD; from 2 weeks to 6 months post-stroke) [41] and hip (aBMD; from 1 week to 12 months post-stroke) [42]. The reason why the level of independent walking function (FAC score) was not correlated with the change in estimated failure load in our study may be that most of our participants with stroke (85%) had already regained independent walking at initial assessment (T1) (refer to Table 1). For community-dwelling individuals with stroke, our findings suggest walking speed is a more valuable assessment of walking status, which is predictive of bone quality.

In a previous study of people with chronic stroke, Lam et al. also attempted to identify clinical factors associated with the change in tibia epiphysis over 1 year. They found that a more severe decline in trabecular vBMD of the distal tibia (measured by pQCT) was associated with poorer baseline quadriceps strength (*ρ* = 0.45, *p* = 0.048) [7]. As factors affecting bone changes are multifaceted, the multivariable regression used in this study is superior to the univariate correlation analysis used previously for predicting the decline in bone strength.

The association between decline in estimated failure load and the above clinical factors identified points to the potential importance of improving or maintaining bone health post-stroke through increasing the level of physical activity and exercise training to enhance walking function as well as muscle and vascular health. However, only two randomized controlled studies have assessed the effect of exercise or physical activity on bone density and structure after stroke [43]. Using a first-generation pQCT device with lower resolution, a positive effect of structured exercise training on the cortical thickness of the midshaft tibia was found in individuals with chronic stroke [44]. Another study found improvement in hip bone density (measured by DXA) in people with subacute stroke after weightbearing training using a dynamic standing bed [45]. More experimental studies using HR-pQCT are warranted to investigate the therapeutic value of different interventions that target the associated factors identified here.

### Limitations

The generalizability of the results presented herein are limited to individuals with chronic stroke who share similar characteristics with the study participants. Mainly due to safety concerns from Coronavirus disease 2019 (COVID-19), there was an increased dropout rate (28%). In a population with multimorbidity, many factors (e.g., age, stroke duration smoking, alcohol consumption, comorbidities) may affect bone changes after stroke, and a larger sample size is needed to delineate their potential influence.

Precision error and LSC could not be established for the estimated failure load in the current study. To enable the interpretation and comparison of these findings, future studies should consider establishing the short-term precision of the scanner system used to assess bone properties and incorporate these metrics (i.e., precisions error, LSC) for all finite element analysis-derived parameters.

To better understand the influence of stroke on peripheral vascular health, future research is recommended to include measures that assess shifts in blood perfusion within the peripheral extremities in response to physical activity (i.e., active hyperemia). These measures may also provide additional mechanistic insights as to how exercise influences bone health after stroke.

Finally, the primary objective of the study was to assess the decline in bone metrics over time in people with chronic stroke, which may serve as an important valuation or projection of fracture risk demonstrated in comparable studies conducted among other clinical populations (e.g., post-menopausal women [8], elderly with osteopenia or osteoporosis [9]). However, the degree to which compromised bone properties contribute directly to fragility fracture incidence among individuals with chronic stroke remains unknown and will require further research involving larger cohorts with a longer follow-up period.

## Conclusions

In conclusion, there was a significant deterioration in trabecular vBMD, trabecular thickness and estimated bone strength in the paretic distal tibia among people with long-standing stroke. Better lower-limb blood flow, higher walking velocity, better muscle strength, higher physical activity level at baseline, and greater improvement in walking speed at 2-year follow-up, were associated with less decline in estimated failure load. More research with a larger sample is still needed to investigate the relative contribution of each clinical factor to the change in estimated failure load. Intervention programs that target these factors may be viable options for improving bone strength in individuals with chronic stroke. Further research is required to prove this.

## Supplementary Information

Below is the link to the electronic supplementary material.

Supplementary file1 (DOCX 34 KB)

Supplementary file2 (DOCX 84.7 KB)

## Data Availability

The original data of this study can be made available upon request.
