# Supplementary material for: Longitudinal bone loss in the paretic leg and its contributing factors in individuals with chronic stroke: a 2-year prospective cohort study
Source: Arch Osteoporos. 2025 Aug 6;20(1):108. doi: 10.1007/s11657-025-01541-4 (PMC12325440; doi:10.1007/s11657-025-01541-4)
Supplement: Supplementary file 1 — Supplementary file1 (DOCX 34 KB) [file 11657_2025_1541_MOESM1_ESM.docx]

### **Title:** [Electronic Supplementary Material (ESM) 1]- Supplemental material for [ Longitudinal Bone Loss in the Paretic Leg and its Contributing Factors in Individuals with Chronic Stroke: A 2-Year Prospective Cohort Study]

**Description:**

Supplemental material- [Electronic Supplementary Material (ESM) 1], for [Longitudinal Bone Loss in Paretic Leg and its Contributing Factors in Individuals with Chronic Stroke: A 2-Year Prospective Cohort Study] by [Huixi OUYANG and Marco YC PANG from the Hong Kong Polytechnic University: marco.pang@connect.polyu.hk] in [ Osteoporosis International]

**ESM 1.1: Samples size estimation.**

**ESM 1.2: Generalized estimating equations results**

### **ESM 1.1: Samples size estimation.**

All sample size estimations were based on an alpha of 0.05 (two-tailed) and a power of 0.8. To address objective 1 (i.e., comparing HR-pQCT data between the stroke and control groups), the sample size estimation was based on a previous study [1] examining the difference in the percentage change in various pQCT variables (i.e., BMC and vBMD) between the two sides in people with chronic stroke, which yielded Cohen’s d values ranging from 0.25 to 1.99 (i.e., equivalent to f = 0.13 to 1.00). Using the smallest effect size (f = 0.13), with two within-subject factors (side [non-paretic vs. hemi-paretic] and time [two assessments]), a sample size of 90 individuals (45 per group) was needed to detect a significant side × time interaction effect.

To address objective 2, generalized estimating equations were used to identify the determinants of bone variables. Previous cross-sectional studies demonstrated R^2^ values ranging from 0.17 to 0.81 (equivalent to effect sizes of f^2^ = 0.20 to 4.26) [2-7]. Using the smallest effect size (f^2^ = 0.20), a sample size of 42 individuals with stroke was needed to detect a significant association between bone variables and other factors.

Taken together, assuming a 15% attrition rate, we aimed to recruit 54 individuals with chronic stroke and 54 control participants for this study.

### **ESM 1.2: Generalized estimating equation analysis results**

There was a significant time effect for total vBMD, cortical vBMD, cortical porosity, bone stiffness, and estimated failure load in both groups (Wald χ^2^= 6.90-44.81, p < 0.01; Tables 4-4 and 4-5). Significant time effects for trabecular area (Wald χ^2^ = 6.6, p = 0.01), cortical area (Wald χ^2^ = 6.08, p = 0.01), and cortical thickness (Wald χ^2^= 9.46, p < 0.01) were only seen in the stroke group, while a significant time effect for trabecular thickness (Wald χ^2^ = 8.68, p < 0.01) was only seen in the control group. There were significant time × side interaction effects for total vBMD (Wald χ^2^ = 5.04, p = 0.025), trabecular vBMD (Wald χ^2^ = 11.05, p < 0.01), trabecular thickness (Wald χ^2^ = 10.59, p < 0.01), and estimated failure load (Wald χ^2^= 4.48, p = 0.03) in the stroke group only.

As shown in Table 4-8 and Table 4-9, the main effect of time was significant for gastrocnemius muscle strength, hallux sensation, and blood flow volume (Wald χ^2^ = 7.65-24.43, p < 0.01) in both the stroke and control groups. Significant time × side interaction effects were only observed for muscle strength (Wald χ^2^ = 4.42, p = 0.036) and blood flow volume (Wald Chi-square = 4.26, p = 0.039) in the control group.

**References**

1. Lam, F., et al., Chronic effects of stroke on hip bone density and tibial morphology: a longitudinal study. Osteoporosis international, 2016. 27(2): p. 591-603.

2. Pang, M.Y.C., M.C. Ashe, and J.J. Eng, Muscle weakness, spasticity and disuse contribute to demineralization and geometric changes in the radius following chronic stroke. Osteoporosis International, 2007. 18(9): p. 1243-1252.

3. Pang, M.Y.C., M.C. Ashe, and J.J. Eng, Tibial Bone Geometry in Chronic Stroke Patients: Influence of Sex, Cardiovascular Health, and Muscle Mass. Journal of Bone and Mineral Research, 2008. 23(7): p. 1023-1030.

4. Pang, M.Y.C., M.C. Ashe, and J.J. Eng, Compromised bone strength index in the hemiparetic distal tibia epiphysis among chronic stroke patients: the association with cardiovascular function, muscle atrophy, mobility, and spasticity. Osteoporosis International, 2009. 21(6): p. 997-1007.

5. Pang, M.Y.C., et al., Relative impact of neuromuscular and cardiovascular factors on bone strength index of the hemiparetic distal radius epiphysis among individuals with chronic stroke. Osteoporosis International, 2012. 23(9): p. 2369-2379.

6. Pang, M.Y.C., F.Z.H. Yang, and A.Y.M. Jones, Vascular Elasticity and Grip Strength Are Associated With Bone Health of the Hemiparetic Radius in People With Chronic Stroke: Implications for Rehabilitation. Physical Therapy, 2013. 93(6): p. 774-785.

7. Yang, F.Z.H. and M.Y.C. Pang, Influence of chronic stroke impairments on bone strength index of the tibial distal epiphysis and diaphysis. Osteoporosis International, 2014. 26(2): p. 469-480.
