# Supplementary material for: Longitudinal bone loss in the paretic leg and its contributing factors in individuals with chronic stroke: a 2-year prospective cohort study
Source: Arch Osteoporos. 2025 Aug 6;20(1):108. doi: 10.1007/s11657-025-01541-4 (PMC12325440; doi:10.1007/s11657-025-01541-4)
Supplement: Supplementary file 2 — Supplementary file2 (DOCX 84.7 KB) [file 11657_2025_1541_MOESM2_ESM.docx]

### **Title:- [**Electronic Supplementary Material (ESM) 2: Figure & Tables**]-** **Supplemental material for [** Longitudinal Bone Loss in the Paretic Leg and its Contributing Factors in Individuals with Chronic Stroke: A 2-Year Prospective Cohort Study**]**

**Description:** Supplemental material- [Electronic Supplementary Material (ESM) 2: Figure &Tables], for [Longitudinal Bone Loss in the Paretic Leg and its Contributing Factors in Individuals with Chronic Stroke: A 2-Year Prospective Cohort Study] by [Huixi OUYANG and Marco PANG from the Hong Kong Polytechnic University: marco.pang@connect.polyu.hk] in [Osteoporosis International]

**ESM Figure 2. 1** Study flowchart

**ESM Table 2.1** Short-term reproducibility and least significant change in bone outcomes measured by HR-pQCT (n=30)

**ESM Table 2.2** Intra-rater and inter-rater reliability of Doppler ultrasound measurements of the popliteal artery for participants with stroke (n=15)

**ESM Table 2.3** Change in modifiable variables over the follow-up period for the stroke and control groups

**ESM Table 2.4** Generalized estimating equations demonstrating the effect of time and side for tibia bone variables in the stroke group (n=46)

**ESM Table 2.5** Generalized estimating equations demonstrating the effect of time and side for tibia bone variables in the control group (n=45)

**ESM Table 2.6** Generalized estimating equations demonstrating the effect of time and side for modifiable variables in the stroke group (n=46)

**ESM Table 2.7** Generalized estimating equations demonstrating the effect of time and side for modifiable variables in the control group (n=45)

**ESM Table 2.8** Item loading for the three-factor rotated solution and communalities for non-modifiable variables and total number of medications (varimax rotation)

**ESM Table 2.9** Item loading for the two-factor rotated solution and communalities for modifiable variables at baseline (varimax rotation)

**ESM Table 2.10** Correlations between the %change in estimated failure load and modifiable, non-modifiable or other variables in the stroke group (n=46)

### **ESM Figure 2.1.** Study flowchart.

Baseline: stroke

(n=64)

Baseline: control

(n=64)

Total dropouts (n=18)

- Covid-19-related safety concerns (n=9)
- secondary stroke (n=3)
- kidney issue (n=1)
- coccyx fracture (n=1)
- lumbar spine dislocation after falling (n=1)
- cancer (n=2)
- left Hong Kong (n=1)

Total dropouts (n=19)

- Covid-19-related safety concerns (n=10)
- left Hong Kong (n=3)
- invalid contact number (n=3)
- abdominal surgery (n=1)
- cancer (n=1)
- knee fracture after falling (n=1)

2-year follow-up: stroke

(n=46)

2-year follow-up: control

(n=45)

Included for analysis: stroke

(n=46)

Included for analysis: control

(n=45)

### **ESM Table 2.1.** Short-term reproducibility and least significant change in bone outcomes measured by HR-pQCT (n=30)

| **Unpublished data** | **Bone variables** | **Tibia precision error (%CV _RMS_)** | **LSC of tibia** | **Norms:**  **male** | **Norms:**  **female** |
| --- | --- | --- | --- | --- | --- |
| **Bone density** | Total vBMD | 0.45% | 1.25% | 0.36% | 2.30% |
|  | Total area | Not established | | 0.46% | -0.11% |
|  | Cortical vBMD | 0.41% | 1.14% | 0.46% | 2.03% |
|  | Trabecular vBMD | 0.65% | 1.80% | 0.94% | 1.32% |
| **Bone morphometry** | Cortical area | 0.89% | 2.47% | -0.10% | 2.17% |
|  | Cortical perimeter | 0.22% | 0.61% | -0.04% | -0.14% |
|  | Cortical thickness | 0.64% | 1.77% | -0.55% | 2.25% |
|  | Trabecular area | 0.27% | 0.75% | -0.01% | -0.43% |
| **Trabecular and cortical microarchitecture** | Trabecular number | 1.44% | 3.99% | 0.75% | 0.64% |
|  | Trabecular thickness | 0.60% | 1.66% | 0.17% | -0.13% |
|  | Trabecular separation | 1.36% | 3.77% | -0.97% | -0.65% |
|  | Trabecular inhomogeneity | 2.23% | 6.18% | -1.37% | -0.70% |
|  | Cortical porosity | 26.61% | 73.71% | -3.68% | -12.65% |
| **Finite element analysis** | Estimated failure load (N) | Not established | | 0.68% | 1.84% |

Abbreviations: HR-pQCT = high-resolution peripheral computed tomography; LSC = least significant change; vBMD = volumetric bone mineral density.

Note: Norms were established based on the change over 2 years in healthy elderly individuals aged 55-65 years.(1) HR-pQCT: high-resolution peripheral computed tomography; LSC: least significant change; vBMD: volumetric bone mineral density. Values are based on unpublished data from 30 cases re-scanned within 1 month. Short-term reproducibility can be expressed as the root-mean-square of the coefficient of variance (CV%_RMS_). Least significant change (LSC) values (calculated from 2.77 × the precision error) are based on the following formula: (2)


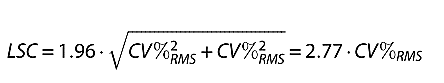


*1. Zhu TY, Yip BH, Hung VW, Choy CW, Cheng KL, Kwok TC, et al. Normative Standards for HRpQCT Parameters in Chinese Men and Women. J Bone Miner Res. 2018;33(10):1889-99.*

*2. Blake GM, Fogelman I. How important are BMD accuracy errors for the clinical interpretation of DXA scans? J Bone Miner Res. 2008;23(4):457-62.*

### **ESM Table 2.2.** Intra-rater and inter-rater reliability of Doppler ultrasound measurements of the popliteal artery for participants with stroke (n=15)

|  |  | Peak systolic velocity (cm/s) | | Diameter  (mm) | | Peak volume flow (mL/min) | |
| --- | --- | --- | --- | --- | --- | --- | --- |
|  |  | P | NP | P | NP | P | NP |
| Inter-rater (ICC 2,3) |  | 0.86 | 0.93 | 0.85 | 0.91 | 0.87 | 0.86 |
| Intra-rater (ICC 3,3) | Rater 1 | 0.94 | 0.96 | 0.95 | 0.96 | 0.90 | 0.96 |
|  | Rater 2 | 0.98 | 0.96 | 0.97 | 0.95 | 0.95 | 0.95 |

Abbreviations: P= paretic side; NP=non-paretic side; ICC= intraclass correlation coefficient.

### **ESM Table 2.3.** Changes in modifiable variables over the follow-up period in the stroke and control groups

|  | **Stroke group (n=46)** | |  | **Control group(n=45)** | |  | | |
| --- | --- | --- | --- | --- | --- | --- | --- | --- |
|  | **Baseline** | **2-Y follow up** | *p* | **Baseline** | **2-Y follow up** | | | *p* |
| Gastrocnemius strength (P, Nm) ^a^ | 41.02±15.89 | 44.31±18.24 | 0.027 | 75.61±36.65 | 98.22±42.60 | | **<0.001** | |
| Gastrocnemius strength (NP, Nm) | 65.53±23.23 | 72.41±28.58 | 0.019 | 75.27±34.15 | 104.83±40.98 | | **<0.001** | |
| Peak systolic velocity (P, cm/s) | 47.62±14.64 | 50.70±12.97 | 0.102 | 44.19±7.61 | 48.40±10.00 | | **0.003** | |
| Blood flow volume (P, mL/min) ^a^ | 37.22±22.08 | 60.58±41.70 | **<0.001** | 28.64±12.67 | 78.74±39.88 | | **<0.001** | |
| Arterial diameter (P, cm) | 0.53±0.12 # | 0.57±0.10 # | **0.005** | 0.52±0.10 | 0.62±0.08 | | **<0.001** | |
| Peak systolic velocity (NP side, cm/s) | 45.36±13.07 | 50.32±14.34 | **0.004** | 44.94±9.04 | 49.70±9.19 | | **0.003** | |
| Blood flow volume (NP, mL/min) ^a^ | 35.45±25.28 | 60.58±37.88 | **<0.001** | 32.23±15.96 | 72.81±42.98 | | **<0.001** | |
| Arterial diameter (NP side, cm) | 0.59±0.12 | 0.62±0.11 | **<0.001** | 0.53±0.09 | 0.62±0.07 | | **<0.001** | |
| Hallux light touch sensation (P, Max:6.65) ^a^ | 3.90±1.14 | 4.62±1.43 | **0.002** | 3.26±0.63 | 3.89±0.72 | | **<0.001** | |
| Hallux light touch sensation (NP, Max:6.65) ^a^ | 3.35±0.93 | 4.01±0.89 | **<0.001** | 3.19±0.64 | 4.05±0.84 | | **<0.001** | |
| 10-meter walk test (m/s) | 0.80±0.39 | 0.86±0.48 | 0.045 | 1.93±0.27 | 2.38±0.54 | | **<0.001** | |
| Physical Activity Scale of the Elderly (Max:400) | 120.91±79.16 | 101.55±76.97 | 0.061 | 148.26±82.16 | 147.43±72.06 | | 0.928 | |
| Composite Spasticity Scale - lower limb total (Max:16) | 7.33±2.43 | 8.78±2.56 | **<0.001** | - | - | | - | |
| Fugl-Meyer Assessment - lower limb (Max:34) | 26.65±4.71 | 25.13±4.68 | **0.001** | - | - | | - | |

Abbreviations: P = paretic side; NP = non-paretic side

Note: Mean ± SD. #: *p*<0.0125 (between-side comparison: paired t test); ^a^: non-parametric test.

### **ESM Table 2.4.** Generalized estimating equations demonstrating the effect of time and side for tibia bone variables in the stroke group (n=46)

|  | **Main Effect** | | | | **Interaction** | |
| --- | --- | --- | --- | --- | --- | --- |
|  | **Time** |  | **Side** |  | **Side** × **Time** | |
| Bone variables | Wald Chi-square | *p* | Wald Chi-square | *p* | Wald Chi-square | *p* |
| Total vBMD (mg HA/cm^3^) | 20.45 | <0.001 | 53.22 | <0.001 | 5.04 | 0.025 |
| Total area (mm^2^) | 5371.56 | <0.001 | 4.34 | 0.037 | 14.11 | <0.001 |
| Trabecular area (mm^2^) | 6.60 | 0.010 | 3.55 | 0.059 | 2.16 | 0.142 |
| Trabecular vBMD (mg HA/cm^3^) | 1.51 | 0.220 | 6.99 | 0.008 | 11.05 | 0.001 |
| Trabecular number (1/mm) | 0.03 | 0.874 | 0.22 | 0.636 | 0.59 | 0.443 |
| Trabecular thickness (mm) | 1.12 | 0.290 | 3.47 | 0.062 | 10.59 | 0.001 |
| Trabecular separation (mm) | 1.81 | 0.178 | 0.93 | 0.336 | 0.10 | 0.751 |
| Trabecular inhomogeneity (mm) | 3.18 | 0.075 | 0.37 | 0.542 | 0.21 | 0.644 |
| Cortical area (mm^2^) | 6.08 | 0.014 | 83.57 | <0.001 | 2.62 | 0.105 |
| Cortical vBMD (mg HA/cm^3^) | 20.47 | <0.001 | 60.53 | <0.001 | 0.36 | 0.550 |
| Cortical perimeter (mm) | 3.22 | 0.073 | 5.43 | 0.020 | 3.55 | 0.060 |
| Cortical porosity (%) | 8.13 | 0.004 | 4.48 | 0.034 | 0.43 | 0.512 |
| Cortical thickness (mm) | 9.46 | 0.002 | 63.94 | <0.001 | 0.27 | 0.603 |
| Failure load (N) | 47.82 | <0.001 | 57.57 | <0.001 | 4.48 | 0.034 |

### **ESM Table 2.5.** Generalized estimating equations demonstrating the effect of time and side for tibia bone variables in the control group (n=45)

|  | **Main Effect** | | | | **Interaction** | |
| --- | --- | --- | --- | --- | --- | --- |
|  | **Time** |  | **Side** |  | **Side** × **Time** | |
| Bone variables | Wald Chi-square | *p* | Wald Chi-square | *p* | Wald Chi-square | *p* |
| Total vBMD (mg HA/cm^3^) | 6.90 | 0.009 | 0.06 | 0.802 | 1.44 | 0.230 |
| Total area (mm^2^) | 4691.64 | <0.001 | 0.16 | 0.692 | 1.06 | 0.304 |
| Trabecular area (mm^2^) | 0.16 | 0.685 | 0.31 | 0.576 | 2.23 | 0.136 |
| Trabecular vBMD (mg HA/cm^3^) | 0.01 | 0.939 | 0.50 | 0.482 | 1.02 | 0.313 |
| Trabecular number (1/mm) | 1.06 | 0.303 | 4.67 | 0.031 | 0.34 | 0.562 |
| Trabecular thickness (mm) | 8.68 | 0.003 | 0.03 | 0.865 | 1.28 | 0.257 |
| Trabecular separation (mm) | 3.32 | 0.069 | 3.43 | 0.064 | 1.31 | 0.253 |
| Trabecular inhomogeneity (mm) | 2.13 | 0.145 | 2.54 | 0.111 | 0.80 | 0.370 |
| Cortical area (mm^2^) | 0.09 | 0.769 | 0.52 | 0.469 | 2.24 | 0.135 |
| Cortical vBMD (mg HA/cm^3^) | 23.67 | <0.001 | 0.00 | 0.951 | 2.24 | 0.135 |
| Cortical perimeter (mm) | 1.85 | 0.174 | 0.82 | 0.364 | 1.04 | 0.307 |
| Cortical porosity (%) | 16.96 | <0.001 | 3.67 | 0.055 | 0.15 | 0.695 |
| Cortical thickness (mm) | 2.19 | 0.139 | 1.46 | 0.228 | 0.59 | 0.444 |
| Failure load (N) | 37.03 | <0.001 | 0.39 | 0.532 | 1.30 | 0.254 |

**ESM Table 2.6.** Generalized estimating equations demonstrating the effect of time and side for modifiable variables in the stroke group (n=46)

|  | **Main Effect** |  |  |  | **Interaction** | |
| --- | --- | --- | --- | --- | --- | --- |
|  | **Time** |  | **Side** |  | **Side** × **Time** | |
|  | Wald Chi-square | *p* | Wald Chi-square | *p* | Wald Chi-square | *p* |
| Gastrocnemius muscle strength (Nm) | 8.41 | 0.004 | 57.44 | <0.001 | 1.12 | 0.290 |
| Gastrocnemius stiffness (N/m) | 0.78 | 0.379 | 1.72 | 0.189 | 0.00 | 0.947 |
| Blood flow velocity (cm/s) | 7.65 | 0.006 | 1.22 | 0.270 | 1.01 | 0.314 |
| Blood flow volume (mL/min) | 24.43 | <0.001 | 0.08 | 0.776 | 0.10 | 0.751 |
| Diameter (cm) | 17.99 | <0.001 | 40.95 | <0.001 | 0.01 | 0.923 |
| Hallux sensation | 18.89 | <0.001 | 24.23 | <0.001 | 0.10 | 0.753 |

**ESM Table 2.7.** Generalized estimating equations demonstrating the effect of time and side for modifiable variables in the control group (n=45)

|  | **Main Effect** | | |  | **Interaction** | |
| --- | --- | --- | --- | --- | --- | --- |
|  | **Time** |  | **Side** |  | **Side** × **Time** | |
|  | Wald Chi-square | *p* | Wald Chi-square | *p* | Wald Chi-square | *p* |
| Gastrocnemius muscle strength (Nm) | 59.02 | <0.001 | 4.39 | 0.036 | 4.42 | 0.036 |
| Gastrocnemius stiffness (N/m) | 0.63 | 0.428 | 0.54 | 0.465 | 0.49 | 0.486 |
| Blood flow velocity (cm/s) | 14.07 | <0.001 | 2.06 | 0.152 | 0.14 | 0.711 |
| Blood flow volume (mL/min) | 91.51 | <0.001 | 0.23 | 0.629 | 4.26 | 0.039 |
| Diameter (cm) | 137.05 | <0.001 | 1.61 | 0.205 | 1.38 | 0.240 |
| Hallux sensation | 44.72 | <0.001 | 2.33 | 0.127 | 2.33 |  |

**ESM Table 2.8.** Item loading for the three-factor rotated solution and communalities for non-modifiable variables and total number of medications (varimax rotation)

|  | Demographic variables | PC1 | PC2 | PC3 |
| --- | --- | --- | --- | --- |
| Factor 1 | Age | **0.757** | 0.341 | -0.078 |
|  | Sex | **0.704** | -0.225 | 0.279 |
|  | Smoking history | **-0.640** | 0.083 | 0.37 |
| Factor 2 | Stroke duration | 0.201 | **0.862** | 0.022 |
|  | Alcohol history | -0.496 | **0.739** | 0.026 |
| Factor 3 | Total number of medications | -0.021 | 0.024 | **0.917** |

Note: Relevant item loadings in bold; Retraction method: principal component (PC); Kaiser-Meyer-Olkin (KMO)=0.505.

**ESM Table 2.9.** Item loading for the two-factor rotated solution and communalities for modifiable variables at baseline (varimax rotation)

|  | Clinical variables at baseline | PC1 | PC2 |
| --- | --- | --- | --- |
| Factor 1 | Gait velocity | **0.877** | 0.033 |
|  | Muscle strength | **0.721** | 0.153 |
|  | Physical Activity Scale for the Elderly | **0.635** | -0.043 |
| Factor 2 | Blood flow volume | 0.050 | **0.991** |

Note: Relevant item loadings in bold; Retraction method: principal component (PC); Kaiser-Meyer-Olkin (KMO)=0.521.

**ESM Table 2.10.** Correlations between the %change in estimated failure load and modifiable, non-modifiable or other variables in the stroke group (n=46)

|  | **Estimated failure load (% change)** | |
| --- | --- | --- |
|  | **r** | ***p*** |
| **Non-modifiable and other variables** |  |  |
| Age | 0.01 | 0.969 |
| Stroke duration | 0.36 | **0.013*** |
| Total number of medications | 0.05 | 0.735 |
| Antihypertensive agents | 0.17 | 0.274 |
| Anticoagulants | 0.26 | **0.080** |
| Hypolipidemic agents | 0.18 | 0.223 |
| Proton pump inhibitors | 0.16 | 0.302 |
| Total number of comorbidities | -0.02 | 0.881 |
| Hypertension | 0.02 | 0.905 |
| Hyperlipidemia | -0.12 | 0.431 |
| Diabetes Mellitus | 0.13 | 0.390 |
| Body Mass Index | 0.24 | 0.117 |
| Sex | -0.32 | **0.030*** |
| Smoking history | 0.14 | 0.352 |
| Alcohol history | 0.02 | 0.898 |
| **Baseline modifiable variables** |  | |
| Physical Activity Scale for the Elderly | 0.27 | **0.071** |
| Gait velocity | 0.30 | **0.043*** |
| Gastrocnemius strength | 0.28 | **0.059** |
| Composite Spasticity Scale | -0.08 | 0.620 |
| Fugl-Meyer Assessment - lower limb | 0.22 | 0.134 |
| Blood flow volume | 0.36 | **0.013*** |
| Artery diameter | 0.06 | 0.705 |
| Hallux light touch sensation | 0.01 | 0.936 |
| Gastrocnemius stiffness | -0.05 | 0.724 |
| Note: *p* value in bold: *p*<0.1, * *p*<0.05. Relative change=(T2-T1)/T1. | | |
